# Affinity Ultrafiltration Mass Spectrometry for Screening Active Ingredients in Traditional Chinese Medicine: A Review of the Past Decade (2014–2024)

**DOI:** 10.3390/molecules30030608

**Published:** 2025-01-30

**Authors:** Yuqi He, Xinyan Zhao, Muze Yu, Di Yang, Lian Chen, Ce Tang, Yi Zhang

**Affiliations:** 1 State Key Laboratory of Southwestern Chinese Medicine Resources, School of Pharmacy, Chengdu University of Traditional Chinese Medicine, Chengdu 611137, China; 18502828016@163.com (Y.H.); 19946734998@163.com (X.Z.); 18285699822@163.com (D.Y.); 2 School of Ethnic Medicine, Chengdu University of Traditional Chinese Medicine, Chengdu 611137, China; 13619090682@163.com (M.Y.); chenlian@stu.cdutcm.edu.cn (L.C.)

**Keywords:** affinity ultrafiltration, AUF-LC-MS technology, traditional Chinese medicine (TCM), active ingredient screening, target

## Abstract

Discovering targets in natural products is a critical and challenging task in new drug development. Rapid and efficient screening of active ingredients from complex systems like traditional Chinese medicine (TCM) is now crucial in drug research. Affinity ultrafiltration (AUF) technology is widely used to screen active ingredients in natural medicines. AUF-liquid chromatography–mass spectrometry (AUF-LC-MS) leverages the affinity between natural medicine extracts and targets to isolate active ingredients from complex matrices, employing LC-MS for detection and activity assessment. This review discusses the developments in employing AUF-LC-MS to analyze TCM and TCM compound preparations over the last decade. This review succinctly presents the advantages and limitations of AUF-LC-MS, illustrating its benefits through the example of screening for active ingredients in natural pharmaceuticals.

## 1. Introduction

Traditional Chinese medicine (TCM), a remarkable medical resource with a long history, has unique advantages in preventing and treating various diseases, particularly in the control of major epidemics and clinical treatment. Approximately 35% of the global pharmaceutical market annually derives directly or indirectly from natural products, predominantly from plant sources (25%), with microbial sources (13%) and animal sources (3%) following [1]. The active ingredients of TCM form the material basis for its therapeutic effects and serve as an important source of biologically active compounds. However, TCM and its formulations often contain numerous chemical components, and the complexity of these mixtures makes the evaluation and identification of active ingredients highly challenging. Thus, identifying the active ingredients in TCM is a critical scientific challenge in its modernization and a significant bottleneck in its global development.

The traditional strategy for researching active ingredients in TCM involves “chemical extraction and separation, molecular structure identification, and pharmacological activity evaluation” [2]. Although effective, this strategy is cumbersome and time-consuming, making it challenging to efficiently screen active structures. Modern pharmacological research indicates that a drug’s affinity for biological macromolecules is the first step in its mechanism of action, and the drug target is the critical starting point for its therapeutic effects in vivo [3]. Small molecules in TCM regulate biological processes and exert medicinal effects by interacting with target proteins in organisms. Consequently, molecular targeting methods for drug screening, based on disease-related biomacromolecules, have emerged.

**Figure 1:**
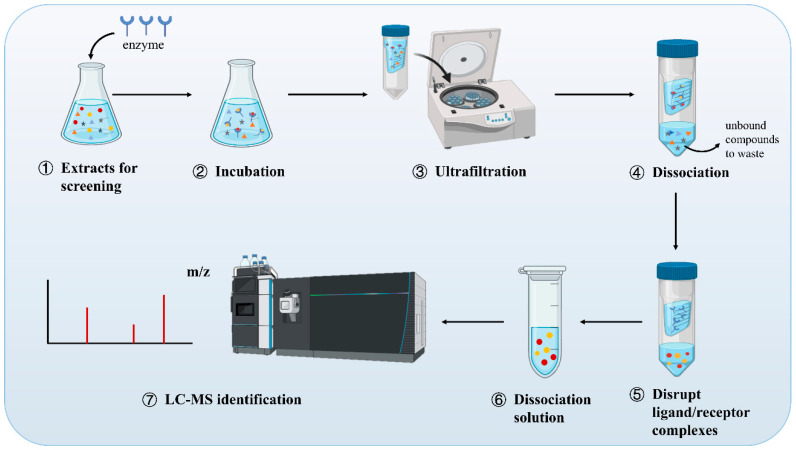
AUF operation flow chart.

Affinity ultrafiltration (AUF)–liquid chromatography (LC)–mass spectrometry (MS) is a solution affinity selection platform that separates target–ligand complexes in solution via ultrafiltration. It serves as a powerful tool for identifying active molecules within complex natural products. Compared with traditional methods, AUF is simple to operate, and it significantly reduces screening time and lowers the consumption of samples and reagents. The technology enables the online integration of various detection instruments, allowing for an accurate reflection of the interaction between the natural conformation of active substances and receptors. Due to its high sensitivity and strong selectivity, AUF-LC-MS holds unique value in small-molecule drug discovery and has garnered widespread attention from the pharmaceutical community. Before that, Chen et al. [4] also provided an overview, summary, and outlook on AUF-LC-MS technology. On this basis, this review provides a more comprehensive review of the basic principles, characteristics, and influencing factors of AUF-LC-MS technology, and summarizes its application in the screening of bioactive components of medicinal plants in the past ten years. For example, *Panax ginseng* has many functions such as enhancing immunity, anti-fatigue, and antioxidants. *Panax ginseng* is rich in saponins, which have a wide range of benefits for the human body. Modern pharmacological research shows that the most important ones are ginsenosides Rg1, Re, and Rb1 [5,6,7]. In recent years, researchers have used α-glucosidase, acetylcholinesterase, Monoamine oxidase type-B, and *N*-methyl-D-aspartic acid as targets, and adopted AUF-LC-MS technology to screen out 24, 16, 7, and 3 active ingredients, respectively [8,9]. In addition, 5, 12, and 32 active ingredients were also screened from *Coptis chinensis* Franch, *Salvia miltiorrhiza* Bge., *Curcuma longa*, etc. [10,11]. Please refer to Figure 1 for specific contents, which provide a certain scientific basis for rapid targeted screening of active ingredients in medicinal plants. This review also discusses the adaptability of this technology to a wider range of natural products and its combination with other analytical techniques, and prospects for its development, so that AUF technology can be widely used internationally.

## 2. AUF-MS: An Overview

AUF combines affinity capture with ultrafiltration, facilitating high-throughput compound screening [12]. Developed in 1981, this technique initially evaluated ultrafiltration’s theoretical and experimental applications in clinical serum binding assays [13]. Discovering drug target proteins is crucial for drug research [14]. In the late 1990s, AUF became widely used in targeted drug discovery and an indispensable tool for many pharmaceutical companies. Over the past decade, significant advancements have been made in AUF in terms of membrane materials, separation properties, and system optimization. Many new affinity membrane materials have been developed recently to enhance the selectivity and performance of the membranes. The separation capabilities of these membranes are enhanced by introducing various affinity ligands or through surface modifications. For instance, the use of hydrophilic polymers, nanomaterials, or composite materials enhances the affinity and anti-fouling properties of these membranes [15,16,17]. To enhance their separation performance, researchers have improved the separation abilities of AUF membranes by combining various affinity ligands, such as different antibodies, proteins, or small molecules. Particularly in complex biological systems, this multi-level separation significantly enhances the purity and efficiency of target molecule separation [18]. Additionally, AUF technology has increasingly adopted automation and intelligent control systems to enhance operational efficiency. For example, the use of real-time sensors to monitor membrane status, in combination with machine learning algorithms for automatic adjustments, enhances both the performance and the operational ease of the membrane system [19]. The ultrafiltration screening method is based on ligand–receptor-specific binding, with screening potential active ligands binding to the target protein by disease-specific characteristics [20]. First, the ligand mixture is combined with the receptor. After ultrafiltration, the ligand dissociates from the receptor, or the binding part is directly observed. Finally, the potential active ingredients are analyzed by LC-MS. AUF-MS is mainly divided into centrifugal ultrafiltration-MS (CU-MS) and pulsed ultrafiltration-MS (PU-MS). In both methods, the basic principle of small-molecule screening is the same: ligand enrichment is achieved through the selectivity of a semi-permeable membrane.

**Figure 2:**
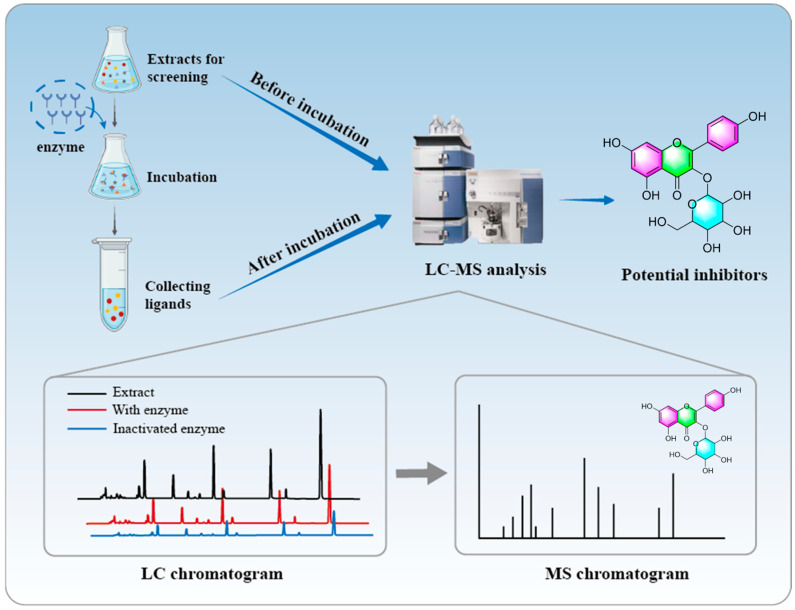
Schematic drawing of AUF-LC-MS screening of potential active ingredients.

The CU-MS by ultrafiltration chamber and LC-MS platform operate independently, necessitating manual injection of ultrafiltration samples into the LC-MS system, hence the term “off-line ultrafiltration”. CU-MS employs commercial ultrafiltration centrifuge tubes to screen compounds, offering straightforward procedures and good reproducibility. Chen et al. [21] developed an off-line ultrafiltration-LC-MS platform to screen for inhibitors of α-glucosidase and pancreatic lipase. Fifteen potential ligands, including glucomoringin, 3-caffeoylquinic acid, and quinic acid, were quickly screened and identified from *Moringa oleifera* leaf extracts. The study identified 14 potential α-glucosidase ligands and 10 potential pancreatic lipase ligands. Feng et al. [22] captured 12 phytochemicals with varying affinities for topoisomerase I, topoisomerase II, COX-2, and ACE2 from *Dysosma versipellis* root and stem extracts by using an off-line ultrafiltration-LC-electrospray ionization (ESI)-MS/MS model. In vitro antiproliferation tests demonstrated that podophyllotoxin and quercetin had the strongest inhibition rates on A549 and HT-29 cells, whereas kaempferol exhibited a significant dose-dependent effect on COX-2. Additionally, quercetin exhibited a strong inhibitory effect on ACE2 (Figure 2).

PU-MS consists of a flow chamber, a magnetic stirrer, and an ultrafiltration membrane [23]. It is an online combination of PU and electrospray MS. After the test sample and target protein are added to the flow chamber, the ligand–receptor complex and inactive components can be separated by applying pressure. Unlike CU-MS, this technology is an online affinity MS screening method. Hence, it is also referred to as online ultrafiltration. PU-MS was first proposed by van Breemen et al. [24] to screen potential compounds binding to target receptors from complex systems. Adenosine deaminase inhibitors were successfully identified from a combinatory chemical library of 20 adenosine analogs by using this method. Beverly et al. [25] utilized PU-MS to evaluate a 35 μL binding chamber’s ability to screen ligands forming noncovalent complexes with protein targets. They found that the platform quickly screened and enriched the carbonic anhydrase inhibitor acetazolamide from bacterial fermentation broth extracts, completing the process in 5 min only.

Compared with PU, CU cannot be integrated with MS online. Additionally, the concentration polarization during centrifugal ultrafiltration can reduce the filtration speed, and in severe cases, cause protein adsorption and deposition on the membrane surface, affecting free drug transport. Thus, CU is primarily used for screening small-molecule active compounds within a limited range. By contrast, PU, which easily integrates with LC-MS to form an automated, high-throughput system, is more effective for describing receptor–ligand binding characteristics, drug metabolism, and product identification. In conclusion, the advantage of ultrafiltration-based methods lies in their ability to rapidly provide binding information between drug targets and compounds. These methods can be used to study the synergistic or antagonistic effects of multiple compounds.

The history of MS traces back to the early 20th century with the invention of the parabolic mass spectrometer by J.J. Thomson. In 1919, Aston developed the first velocity-focusing MS, marking a significant milestone in the field. Initially, MS was primarily used to determine the atomic weight of elements and isotopes. With advancements in ion optics theory, the technology continually improved, and by the late 1950s, it was widely applied in the analysis of inorganic and organic compounds. Owing to its high sensitivity, accuracy, and resolution, MS has become one of the most crucial analytical techniques in life sciences, medicine, and chemistry [26]. The advent of MS technology, particularly soft ionization methods like ESI and matrix-assisted laser desorption/ionization (MALDI), has extended the application of MS to the early stages of drug discovery, specifically in the identification of lead compounds [27]. Compared with earlier detection methods, MS does not require derivatization or isotope labeling, thereby expanding the range of applicable compounds, accelerating detection, and enhancing sensitivity and specificity. Thus, integrating MS with target affinity techniques—referred to as target molecule affinity-MS—has made drug screening more efficient and effective. In recent years, numerous MS techniques have been developed to address the increasing demand for analyzing and identifying specific components within complex substrates from multiple perspectives. These include techniques such as AUF-LC-MS, ESI-Q-TOF-MS, ultrahigh-performance LC (UPLC)–Orbitrap–(time-of-flight) TOF-MS, MALDI-TOF-MS, LC-MS, GC-MS, FT-ICR-MS, and DART-MS.

Based on the above explanation, the ultrafiltration method effectively enriches and separates ligands that bind to target proteins while being easy to operate and cost-effective. AUF can screen ligand–protein complexes from unbound substances, and when combined with LC and MS, it enables rapid separation and identification of potential active ingredients. It can identify target substances at various concentrations, and it is suitable for analyzing small quantities of complex mixtures such as combinatorial compound libraries and extracts or fractions of medicinal plants. When AUF is combined with LC-MS^n^, the high sensitivity of MS compensates for the limitations of LC in detecting minute components with low sample content [28]. As a high-throughput method, AUF-LC-MS performs well in screening active substances without stringent sample size requirements and offers additional advantages such as simplicity of operation and strong specificity. However, this method has certain limitations: false positives resulting from nonspecific adsorption in the ultrafiltration process typically need to be addressed through parallel control experiments using an inactivated target protein group or a serum protein replacement group. Additionally, ultrafiltration screening is primarily based on the affinity between the target protein and the ligand. As a result, while it evaluates the ligand’s affinity for the target protein, it does not directly reflect the ligand’s biological activity [29].

## 3. Advantages and Characteristics of AUF-LC-MS

Currently, various methods exist for screening active ingredients, such as cell membrane chromatography, magnetic bead screening, UV-visible spectroscopy, nuclear magnetic resonance (NMR), fluorescence, and electrochemical methods (Table 1) [30,31,32,33,34,35]. Compared with these methods, the combination of AUF and MS for screening small-molecule active substances in TCM offers several advantages, including ease of operation, high sensitivity, and specific results. Traditional chromatographic methods based on optical or radioactive substances often encounter matrix interference [36]. This interference complicates the identification and analysis of complex components in natural products. For instance, UV-visible spectroscopy measures α-glucosidase activity by hydrolyzing *p*-nitrophenyl-*α*-*D*-glucopyranoside, producing *p*-nitrophenol, detectable at 400 nm [37]. However, NMR is time-consuming and not well suited for rapid inhibitor screening. Additionally, fluorescence and electrochemical methods suffer from significant interference issues [38,39]. Consequently, a rapid and accurate method to screen active compounds with inhibitory effects is urgently needed. The ligand matrix does not affect the screening process of affinity MS. Thus, this unique advantage renders it particularly suitable for screening active ingredients in complex systems, especially traditional medicinal plants.

Owing to its high sensitivity and selectivity, AUF-LC-MS has been effectively utilized to isolate and identify target substances from complex samples, playing a pivotal role in extracting active molecules from natural products. In AUF, researchers study the interactions between small drug molecules and biological targets in solution. Binding between AUF receptors and ligands occurs in solution, which avoids alterations in their properties from labeling or chemical coupling to solid supports, thereby preserving their natural conformation and interactions. Ultrafiltration requires only small quantities of the target, and some protein targets can be reused, making it a viable option when targets are costly, scarce, or available in limited quantities [44]. Alternatively, the retention capability of the ultrafiltration membrane allows for the direct selection of active components that bind to target substances without the need for pretreatment, such as in immobilized enzyme online MS and cell membrane chromatography-MS [45,46]. AUF-MS enables rapid determination of binding constants between biological targets and small drug molecules while concurrently providing activity data for these molecules. In the combined AUF-MS approach, AUF exhibits robust specificity and screening capabilities for small ligands in complex mixtures. Meanwhile, LC-MS offers potent functionality for efficient separation and structural identification, effectively minimizing matrix interference.

## 4. Factors Affecting AUF Screening

**Figure 3:**
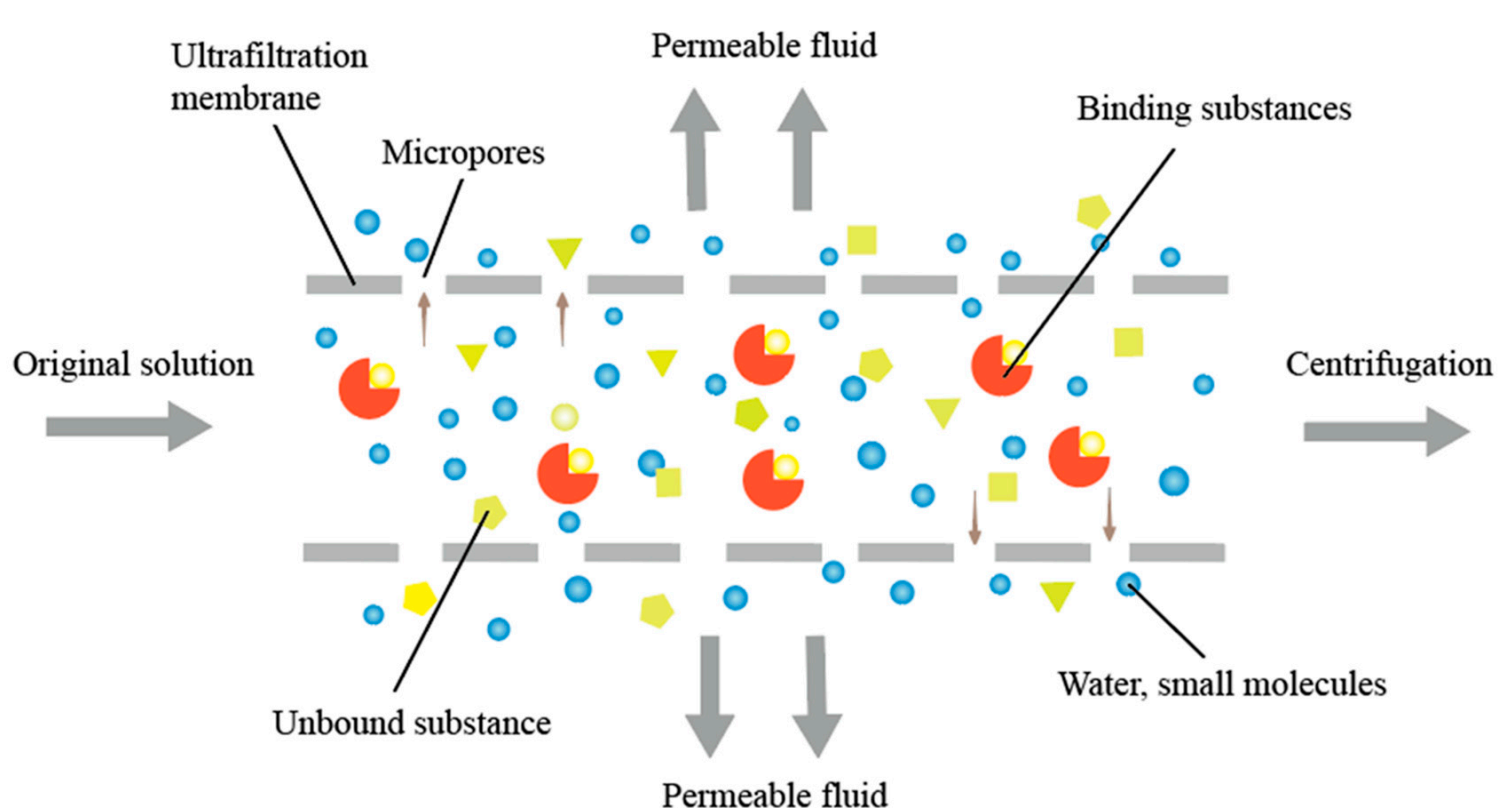
Ultrafiltration membrane filtration principle diagram.

AUF-LC-MS is extensively utilized for screening active ingredients from complex substrates because of its high-throughput capabilities. However, this technique has limitations, including the possibility that some identified candidates may not exhibit the expected activity or may show elevated activity, leading to potential false positives [47]. Various factors must be considered during the experimental process, including the concentration of the target and screening substances, the material of the ultrafiltration membrane, the selection of the dissociation solvent, the interception volume, the co-incubation time, the centrifugal speed, and the solution pH, to mitigate false-positive or false-negative results. The screening conditions must be optimized to ensure the high efficiency and specificity of the screening results, and operations should be rationally designed and standardized. Additionally, the design of negative control experiments is crucial for reducing false positives and improving the accuracy of the results (Figure 3).

### 4.1. Concentration of the Target and the Screened Substances

The concentrations of targets and screening substances are critical factors influencing the affinity filtration process. If the ligand concentration is significantly higher than that of the target protein, it may prevent some active ingredients from binding to the target proteins because ligand binding to target proteins is inherently competitive, leading to false negative results. Conversely, if the ligand concentration is too low, it may enhance nonspecific adsorption, thus increasing the likelihood of false positives. These false positives are often due to the nonspecific binding of the compound to the target protein. Yang et al. [48] were the first to verify AUF-LC screening results to eliminate false positives by using competitive binding experiments. In fact, competitive binding experiments not only eliminate false positives but also exclude ligands that bind to different sites than those of competitively binding compounds. Wang et al. [47] evaluated the feasibility of using competitive binding experiments combined with AUF-LC to identify xanthine oxidase (XOD) inhibitors in *Perilla frutescens* (L.) Britt., aiming to reduce false positives. In the experiment, *P. frutescens* extracts were incubated with XOD-free, XOD-present, or XOD-blocked active sites before ultrafiltration, and the total binding degree and specific binding degree of each compound were calculated on the basis of peak area. The results indicated that AUF-LC significantly reduced the number of false positives identified. However, this method cannot eliminate all false positives and may exclude some effective inhibitors.

Therefore, a thorough methodological review is essential to obtain reliable binding results. The equilibrium dissociation constant (KD) is a critical metric for evaluating the interaction between a ligand and its target protein, with each component having its own distinct KD value. The KD values of the receptor and target ligand should be closely matched; otherwise, significant discrepancies may result in false positives or false negatives. In general, the receptor concentration should be close to the KD value of the weakest ligand. If the ligand concentration is too high, only ligands with strong binding affinity could bind to the target protein at competitive binding sites. Therefore, in actual experiments, the ligand concentration should be equal to or less than that of the receptor. Wang et al. [49] developed an AUF-UPLC method to directly determine the KD of compounds in *P. frutescens* extracts and their target proteins, including the KD determination for α-glucosidase ligands in the ethyl acetate fraction of *P. frutescens.* The recovery rate, binding degree, and signal-to-noise ratio of α-glucosidase ligands in PFEA were determined using AUF-LC, followed by KD calculation using the proposed equilibrium. Oleanolic acid and apigenin were identified as high-affinity ligands of α-glucosidase, with KDs of 44.9 and 88.5 μM, respectively. These values were consistent with the results from isothermal titration calorimetry, kinetic analysis, and molecular docking simulations. The results demonstrate that this method is simple and easy to implement, allowing direct determination of KD values for compounds in natural product extracts without the need for internal standards or calibration agents. Optimizing these methods can enhance the screening accuracy and reliability of AUF-LC-MS, providing a robust foundation for the identification of active ingredients in complex substrates.

### 4.2. Ultrafiltration Membrane Material

In AUF-LC-MS, ultrafiltration membranes separate ligand–receptor complexes from unbound components. The selection of ultrafiltration membranes primarily involves two factors: pore size and material [40]. An ideal ultrafiltration membrane should effectively retain the target biological macromolecules while preventing leakage or clogging. The pore size should be less than one-third of the biomacromolecule’s size to ensure effective retention [12]. Selecting the appropriate pore size improves separation efficiency and prevents leakage of unbound components. An ideal ultrafiltration membrane material should minimize specific adsorption with potential ligands and receptors. Common ultrafiltration membrane materials include polyvinyl fluoride, polysulfone, polyether ketone, and methylcellulose [50]. These materials exhibit low nonspecific binding and are therefore widely used in ultrafiltration membrane production. Selecting the appropriate pore sizes and materials optimizes the separation efficiency of AUF-LC-MS and enhances the accuracy and reliability of the experiment, ensuring the authenticity of ligand–receptor interactions and reducing false-positive results.

### 4.3. Choice of Dissociation Solvent

The complex components, diverse structures, and varying polarities of TCM extracts make it challenging to successfully dissociate ligands from the affinity target while minimizing nonspecific adsorption, a key factor affecting screening results. Two main methods are currently used to denature enzymes: adding acid to the dissociation solvent to inactivate the enzyme in a low pH environment or using organic solvents for enzyme denaturation.

However, using organic solvent-based dissociation solutions only can sometimes increase nonspecific adsorption. Some related studies have demonstrated that acid-containing organic solvents, as opposed to those with organic solvents only, can effectively reduce nonspecific adsorption of non-affinity interacting substances. For example, Xie et al. [51] used a methanol–water (90:10) mixture to screen potential TCM components targeting 5-lipoxygenase and cyclooxygenase-2. Comparison of the ultrafiltrate chromatograms between the experimental and control groups revealed significant differences in the peak areas of active ingredients, with lower signals for nonspecifically adsorbed substances. Conversely, some researchers have successfully screened small-molecule inhibitors of cyclooxygenase and glutathione reductase from TCM by using dissociation solutions containing organic solvents only [52,53]. The findings indicate that different dissociation solutions yield varying effects, necessitating multiple experimental attempts to optimize dissociation conditions.

In summary, selecting an appropriate dissociation solvent is essential for reducing nonspecific adsorption and enhancing the accuracy of screening results. Multiple experimental attempts are recommended to identify the optimal dissociation conditions by comparing results, thereby effectively screening the active ingredients in TCM extracts.

## 5. Screening Technology and Application

### 5.1. High-Throughput Screening (HTS) of Active Ingredients of TCM

Efficient and rapid screening of active ingredients from complex systems, such as TCM, remains a key challenge in modern pharmaceutical research. Traditional methods of chemical separation, structural identification, and activity screening face the following several issues: unclear objectives, cumbersome procedures, high workload, lengthy processes, and potential loss of active ingredients. Recent pharmacological research has demonstrated that the affinity between drugs and biological macromolecules—such as enzymes, receptors, DNA, and RNA—is crucial for drug action. Molecular targeting strategies for drug screening have emerged, focusing on disease-related biological macromolecules as targets. Ultrafiltration offers excellent separation and minimizes matrix interference, whereas LC-MS provides powerful analytical capabilities for the rapid identification of multiple components. Combining these technologies to discover small-molecule active ingredients in TCM holds significant potential. Recently, this combined approach has been successfully applied to the screening of lead compounds, compound libraries, and active ingredients from natural products.

**Figure 4:**
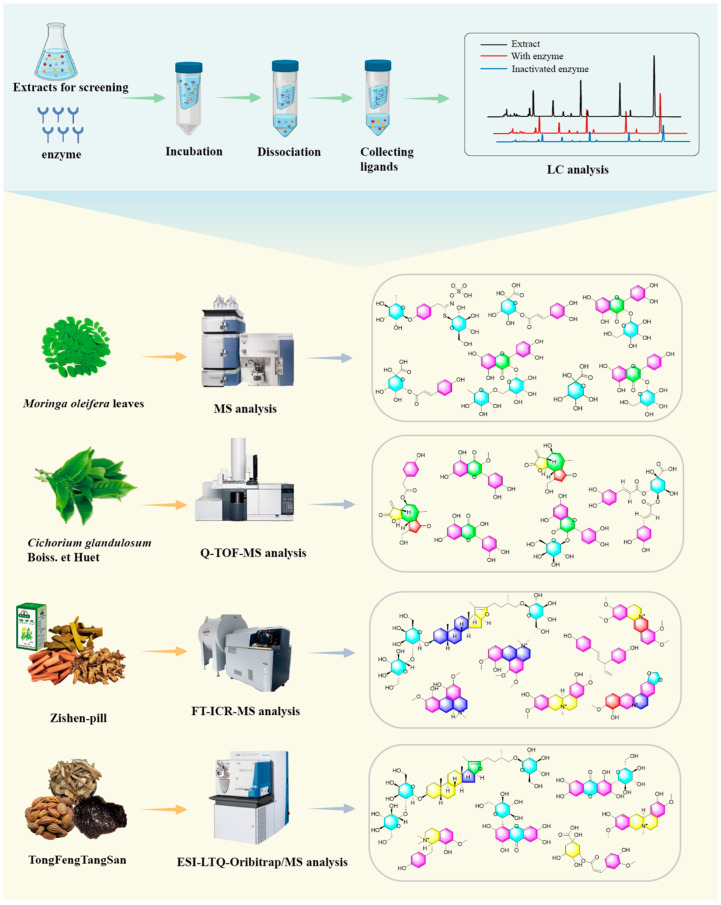
AUF application example diagram.

Numerous studies have confirmed that this method rapidly screens and identifies complex ligands in natural products (Figure 4). In recent years, scientists have frequently combined AUF with MS detection to screen active ingredients in combinatory chemical libraries, identifying novel inhibitors of key targets like α-glucosidase. α-Glucosidase is a key enzyme in carbohydrate hydrolysis, cleaving the α-1,4-glucoside bond at the non-reducing end of oligosaccharides, thereby releasing glucose and raising blood sugar levels. α-Glucosidase inhibitors reduce glucose production by inhibiting this enzyme’s activity, and they are widely used in the treatment of type 2 diabetes mellitus (T2DM) [54]. Although some α-glucosidase inhibitors derived from microorganisms, such as acarbose and voglibose, are used clinically, they can cause severe gastrointestinal side effects [55,56]. Natural α-glucosidase inhibitors from medicinal plants offer potential as alternative treatments for T2DM due to their low toxicity. Consequently, researchers have recently screened potential α-glucosidase inhibitors from various natural plants, including *Cichorium glandulosum* Boiss. et Huet, a chicory species in the Asteraceae family and a traditional Uighur medicinal plant. *C. glandulosum* is listed as a “medicinal food homology” item in the 2015 Catalogue of Homologous Medicine and Food by the National Health and Family Planning Commission of China. Studies have shown that chicory exhibits significant hypoglycemic activity and inhibits α-glucosidase [57,58]. Chen et al. [59] used AUF-LC-MS to screen and identify four potential α-glucosidase inhibitors from *C. glandulosum* seed extract to further investigate its hypoglycemic components. The preliminary identification included esculetin, chlorogenic acid, isochlorogenic acid B, and osochlorogenic acid A. Subsequently, Abudurexiti A et al. [60] used AUF to screen *C. glandulosum* extracts, identifying the following six potential α-glucosidase inhibitors: quercetin, lactucin, 3-*O*-methylquercetin, hyperoside, lactucopicrin, and isochlorogenic acid B. Potential α-glucosidase inhibitors have been screened from various natural plants, including the leaves of *Rubus suavissimus* and *Inonotus obliquus* and the roots of *Siraitia grosvenorii* [61,62,63]. The screening results of α-glucosidase-targeted active ingredients are detailed in Table 2.

Medicinal plants have been widely used to treat various diseases for thousands of years owing to their value as natural resources. Extracting biologically active compounds from medicinal plants has become a major focus of research worldwide. Chemical components in medicinal plants often have low abundance, complex structures, and multiple biological targets. The active ingredients and mechanisms of action are often challenging to define precisely. AUF-LC-MS is well suited for screening active ingredients in complex natural products. This technology combines the separation and analytical strengths of AUF and LC-MS, facilitating HTS and rapid identification of bioactive components in complex natural products. *Andrographis paniculata* (Burm. f.) Wall. ex Nees is derived from the dried aboveground parts of the plant. It exhibits a broad range of pharmacological activities in vivo and in vitro studies, with anti-inflammatory effects being the most prominent. Cyclooxygenase-2 (COX-2) is a key enzyme in prostaglandin (PG) synthesis, and its inhibitors are effective anti-inflammatory agents. Jiao [64] developed an AUF-based analytical method combined with UPLC and quadrupole TOF-MS (BAUF-UPLC-Q-TOF-MS) for rapid screening and identification of COX-2 ligands. Five COX-2 inhibitors were identified from *A. paniculata* extracts. Apart from its anti-inflammatory properties, *A. paniculata* exhibits immunomodulatory and antiviral effects. Feng [65] screened 11 potential ligands from *A. paniculata* targeting COX-2, IL-6, and ACE2.

In addition to the previously mentioned disease-related targets, AUF-MS can be used to screen 24 target active ingredients, including lipase, thrombin, and tyrosinase (TYR). Lipase catalyzes the hydrolysis of fats (lipids). Lipase inhibitors regulate lipids by inhibiting the catalytic activity of human pancreatic lipase, a key enzyme in triacylglycerol hydrolysis, aiding in the control or treatment of obesity-related conditions. TYR is a rate-limiting enzyme in melanin production. Albinism is a genetic disorder caused by mutations in the TYR gene, leading to impaired TYR production. Thrombin (FIIα) is a key enzyme in thrombosis and a downstream component of the coagulation pathway. It converts fibrinogen into fibrin and coagulation factor XIII into factor XIIIα. This process combines with calcium ions to form the fibrin network, a critical step in thrombosis. Consequently, FIIα has gained widespread attention as a target for antithrombotic therapies. Table 2 summarizes the applications of AUF-MS in screening natural product extracts from January 2014 to May 2024.

**Table 2:** List of active ingredients of natural products screened by AUF technology.

Target Protein	Natural Products	Active Ingredients	Ref.
α-Glucosidase	*Panax Ginseng*	Twenty-four compounds	[8]
	*Rhizoma Coptidis*	Jatrorrhizine, epiberberine, coptisine, palmatine, berberine	[10]
	*Moringa oleifera* leaves	Fourteen compounds	[21]
	*Perilla frutescens*	Nine compounds	[49]
	*Cichorium glandulosum*	Esculetin, chlorogenic acid, isochlorogenic acid B, isochlorogenic acid A	[59]
	*Cichorium glandulosum*	Quercetin, lactucin, 3-*O*-methylquercetin, hyperoside, lactucopicrin, isochlorogenic acid B	[60]
	*Rubus suavissimus* leaves	Twenty-six compounds	[61]
	*Inonotus obliquus*	(*E*)-4-(3,4-dihydroxyphenyl) but-3-en-2-one	[62]
	*Siraitia grosvenorii* Roots	Seventeen compounds	[63]
	*Cichorium glundulosum* root	Baicalin, lactupicrin	[66]
	*Trifolium pratense*	Daidzin, ononin, daidzein, genistein, fomononetin, biochanin A	[67]
	*Cyclocarya paliurus* leaves	Mainly damarane-type triterpenoid saponins	[68]
	*Radix Astragali*	Thirteen prototype isoflavonoids and one monohydroxylated metabolic isoflavonoid	[69]
	*Scutellaria baicalensis* Georgi	Baicalin, wogonoside, 5,7,3,2′,6′-pentahydroxy flflavanone, chrysin-6-*C*-arabinosyl-8-*C*-glucoside, chrysin-6-*C*-glucosyl-8-*C*-arabinoside, wogonin	[70]
	*Polygonatum odoratum*	Five phenethyl cinnamides and four homoisoflavanones	[71]
	*Scutellaria baicalensis* Georgi	Baicalein, baicalein, wogonin, chrysin, oroxylin A	[72]
	*Ginkgo biloba*	Eleven compounds	[73]
	*Buddleja* Flos	Thirteen phenylethanoid glycosides and twenty flavonoids	[74]
	*Cercis chinensis*	Twelve compounds	[75]
Cyclooxygenase 2	*Dysosma versipellis*	Nine compounds	[22]
	*Anemarrhenae rhizoma*	Timosaponin A-II, timosaponin A-III, timosaponin B-II, timosaponin B-III, anemarrhenasaponin I	[51]
	*Andrographis paniculata*	Andrographolide, 14-deoxy-11,12-didehydroandrographiside, andrographidine E, andrographidine D, deoxyandrographolide	[64]
	*Andrographis paniculata*	Eleven compounds	[65]
	*Kadsura coccinea*	Twenty-one compounds	[76]
	*Rhamnus davurica*	Vitexin, Taxifolin, Aromadendrin, Kaempferol 7-*O*-glucoside, berberine III, apigenin, kaempferol, rhamnocitrin, sakuranetin, questin, physcion	[77]
	*Trifolium pratense L.*	Rothindin, ononin, daidzein, trifoside, pseudobaptigenin, formononetin, biochanin A	[78]
	*Paris polyphylla*	Polyphyllin I, II, VI, VII	[79]
	*Curcuma longa*	Thirteen compounds	[80]
	*Sinopodophyllum hexandrum*	Rutin, quercetin 3-*O*-glucoside, kaempferol 3-*O*-glucoside, *β*-Apopicropodophyllin, quercetin, isorhamnetin, kaempferol, podophyllotoxin	[81]
	*Saussurea obvallata*	Coniferin, syringin, roseoside, grasshopper ketone	[81]
	*Moutan cortex*	Gallic acid, methyl gallate, galloylpaeoniflflorin, 1,2,3,6-Tetra-*O*-galloyl-*β*-*D*-glucose, 1,2,3,4,6-Penta-*O*-galloyl-*β*-*D*-glucopyranose	[82]
Xanthine oxidase	*Perilla frutescens*	Kaempferol-3-*O*-rutinoside, rosmarinic acid, methyl-rosmarinic acid, apigenin, 4′,5,7-trimethoxyflflavone were identifified, from total eleven compounds	[47]
	*Trifolium pratense*	Daidzin, ononin, daidzein, genistein, fomononetin, biochanin A	[67]
	*Panax japlcus var.*	24(*R*)-majoroside R1, chikusetsusaponin IVa, oleanolic acid-28-*O*-*β*-*D*-glucopyranoside, notoginsenoside Fe, ginsenoside Rb2, ginsenoside Rd	[83]
	Flos *Chrysanthemum*	Luteolin-7-*O*-glucoside, apigenin-7-*O*-glucoside, luteolin, apigenin	[84]
	*Selaginella tamariscina*	Amentoflavone, robustaflavone	[85]
	Celery seeds (*Apium graveolens* L.)	Luteolin-7-*O*-apinosyl glucoside, luteolin-7-*O*-glucoside, luteolin-7-*O*-malonyl apinoside, luteolin-7-*O*-6′-malonyl glucoside, luteolin, apigenin, chrysoeriol	[86]
	the roots of *Lindera reflexa* Hemsl	Pinosylvin, pinocembrin, methoxy-5-hydroxy-*trans*-stilbene	[87]
	*Azadirachta indica*	Carnosic acid	[88]
	*Ligusticum chuanxiong*	Isochlorogenic acid C, senkyunolide I	[89]
	*Curcumae Rhizoma*	Fifteen compounds	[89]
	*Curcuma phaeocaulis* Valeton	Fifteen compounds	[90]
	*Polygonum Amplexicaule*	Gallic acid, procyanidin B2-3″*O*-gallate, 11-*O*-galloylbergenin, (−)-epicatechin gallate, di-galloyl-*O*-bergenin	[91]
	*Salvia miltiorrhiza* Bge.	Seventeen compounds	[92]
Acetylcholinesterase	*Panax ginseng*	Ginsenoside Ro, Rb2, Rg1, Re, Rf, Rb1, Rc, Rb3, Rd, Rs1, Ra6, chikusetsusaponin IVa, gypenoside XVl, compound O, pseudoginseoside Rc1, zingibroside R1	[9]
	*Terminalia chebula* fruits	Mainly gallotannins and ellagitannins	[68]
	*Fibraurea recisa* Pierre.	Twelve compounds	[93]
	*Coptis chinensis* Franch	Columbamine, jatrorrhizine, coptisine, palmatine, berberine	[94]
	*Zanthoxylum nitidum*	Jatrorrhizine, columbamine, skimmianine, palmatine, epiberberine	[94]
	*Azadirachta indica*	*D*-(+)-catechin, (−)-epicatechin, carnosol	[95]
	*Hedyotis diffusa*	Quercetin-3-*O*-sophoroside, quercetin-3-*O*-[2-*O*-(6-*O*-*E*-sinapoyl)-*β*-*D*-glucopyranosyl]-*β*-*D*-glucopyanoside, quercetin-3-*O*-[2-*O*-(6-*O*-*E*-feruloyl)-*β*-*D*-glucopy-ranosyl]-*β*-*D*-glucopyranoside, (*E*)-6-*O*-*p*-coumaroyl scandoside methyl ester	[96]
Topoisomerase I	*Dysosma versipellis*	Twelve compounds	[22]
	*Lycoris radiate*	Hippeastrine, camptothecin	[44]
	*Rhamnus davurica* Pall.	Eleven compounds	[77]
	*Paris polyphylla*	Polyphyllin I, II, VI, VII	[79]
	*Sinopodophyllum hexandrum*	Isocorydine, rutin, quercetin 3-*O*-glucoside, kaempferol 3-*O*-glucoside, *β*-apopicropodophyllin, quercetin, isorhamnetin, kaempferol, podophyllotoxin	[80]
	*Rhamnus davurica*	Aromadendrin, naringeninb, apigenin, quercetina, rhamnocitrinb, sakuranetin, questinb, physcionb	[97]
Arachidonate 5-lipoxygenase	*Saposhnikovia divaricata* (Turcz.) Schischk	Prim-*O*-glucosylcimifugin, 4′-*O*-*β*-*D*-glucosyl-5-*O*-methylvisamminol, cimifugin, sec-*O*-glucosylhamaudol	[98]
	*Smilax glabra* Roxb.	Astilbin, isoastilbin, engelitin, isoengelitin, resveratrol	[98]
	*Pueraria lobata*	puerarin, daidzin, 3′-methoxy-puerarin, 3′-hydroxy-puerarin, daidzein	[98]
	*Carthamus tinctorius*	Hydroxyl safflower yellow A, anhydrosafflor yellow B	[98]
	*Radix Saposhnikoviae* via	Prim-*O*-glucosylcimifugin, cimifugin, 5-*O*-methylvisamminol, sec-*O*-glucosylhamaudol, hamaudol	[99]
Topoisomerase II	*Dysosma versipellis*	Twelve compounds	[22]
	*Paris polyphylla*	Polyphyllin I, II, VI, VII	[79]
	*Sinopodophyllum hexandrum*	Isocorydine, rutin, quercetin 3-*O*-glucoside, quercetin, isorhamnetin, kaempferol	[80]
Augmented reality	*Lysimachia christinae*	1,5-di-hydroxy-1,5-*di*-[(*E*)-3-(4-hydroxyphenyl)-2-propenoic]-3-pentanonyl	[100]
	Peruvian tea plant infusions	Chlorogenic acid, 3,5-*di*-*O*-caffeoylquinic acid, 1,3,5-*tri*-*O*-caffeoylquinic acid	[101]
	*Hypericum laricifolium* Juss.	Protocatechuic acid, chlorogenic acid, caffeic acid, kaempferol 3-*O*-glucuronide, quercetin, kaempferol	[102]
Neuraminidase	*Polygonum cuspidatum*	*Trans*-polydatin, *cis*-polydatin, emodin-1-*O*-*β*-*D*-glucoside, emodin-8-*O*-*β*-*D*-glucoside, emodin	[103]
	*Baphicacanthus cusia*	2,4(1*H*,3*H*)-quinazolinedione, 4(3*H*)-quinazolinone, 2(3*H*)-benzoxazolone, tryptanthrin, indirubin	[104]
	*Angelica pubescens*	Thirteen compounds	[105]
Angiotensin-converting enzyme 2	*Dysosma versipellis*	Twelve compounds	[22]
	*Andrographis paniculata*	Eleven compounds	[65]
	*Sinopodophyllum hexandrum*	Isocorydine, rutin, quercetin 3-*O*-glucoside, kaempferol 3-*O*-glucoside, *β*-apopicropodophyllin, isorhamnetin, kaempferol, podophyllotoxin	[83]
Pancreatic lipase	*Moringa oleifera* leaves	Eleven compounds	[21]
	*Dendrobium officinale*	Vicenin II, isoschaftoside, schaftoside, vitexin 2″-*O*-glucoside, vitexin 2″-*O*-rhamnoside, rutin, isoquercetrin, kaempferol 3-*O*-*β*-*D*-glucopyranoside, naringenine, linolenic acid, palmitic acid	[106]
Protein Tyrosine Phosphatase-1B	*Puerariae Lobatae* Radix	Daidzin, Puerarin	[107]
	Black tea	(−)-epicatechin-3-*O*-gallate, epigallocatechin gallate, positive control	[107]
Estrogen receptor	*Arnebia euchroma*	Twenty-one compounds	[108]
G-quadruplex DNA	*Macleaya cordata*	Protopine, allocryptopine, sanguinarne, chelerythrine	[109]
Superoxide dismutase	*Azadirachta indica*	Gallic acid, protocatechuic acid, (−)-epicatechin	[88]
Lactate dehydrogenase	*Trifolium pratense*	Biochanin A, genistein, fomononetin, ononin	[67]
Monoamine oxidase type-B	*Panax ginseng*	Ginsenoside Rg1, Re, Rb1, Rc, Ro, Rb2, Rd	[9]
*N*-Methyl-D-aspartic acid	*Panax ginseng*	Ginsenoside Rg1, Re, Rf	[9]
Matrix Metallopeptidase 2	*Smilax glabra* Roxb., *Smilax china* L., *Saposhnikovia divaricate* (Turcz.) Schischk	Resveratrol, engelitin, asibinn, 4′-*O*-*β*-*D*-glucosyl-5-*O*-methylvisamminol, cimifugin, prim-*O*-glucosylcimifugin, sec-*O*-glucosylhamaudol	[110]
UDP-glucuronosyltransferase 1A1	Polygonum multiflorum root	*Cis*-2,3,5,4′-tetrahydroxystilbene-2-*O*-*β*-glucoside, *trans*-2,3,5,4′-tetrahydroxystilbene-2-*O*-*β*-*d*-glucoside, emodin-8-*O*-*β*-*d*-glucoside, emodin	[111]
Interleukin-6	*Andrographis paniculata*	Eleven compounds	[65]
Tyrosinase	*Dryopteris crassirhizoma* rhizome	Twenty-two compounds	[112]
Lactate dehydrogenases	*Azadirachta Indica*	Carnosol	[95]
Epidermal growth factor receptor erbB1	*Psoralea Fructus*	Psorachalcone A, psoralen, bakuchalcone	[113]

### 5.2. Screening of Active Ingredients in TCM Compound Preparations

TCM compound preparations are formulated on the basis of TCM theory. Their chemical components are highly complex, making it challenging to rapidly screen and identify active ingredients using conventional analytical methods. Historically, clarifying the bioactive components and mechanisms of action in single medicinal plants has been difficult, let alone in natural drug formulas, due to their low content, complex chemical structures, and multicomponent, multitarget effects. AUF-LC-MS remains one of the most powerful tools for screening active compounds from complex natural products (Table 3).

In recent years, Ronghua Dai’s research group [114] has employed AUF-LC-MS to study the interactions between extracts of Zishen Pills, a TCM compound preparation, and biological target proteins. COX-2 is a key enzyme that catalyzes the conversion of arachidonic acid (AA) into PGs. It is specifically induced during inflammation, degeneration, and tumorigenesis. The research group employed AUF-LC-MS to investigate the interaction between Zishen Pill extract and COX-2, selecting celecoxib and glipizide as positive and negative controls, respectively. The study identified 20 compounds that specifically bind to COX-2, 8 of which are potential COX-2 inhibitors. Their structures were elucidated using Fourier transform ion cyclotron resonance MS. Further validation was conducted using in vitro COX-2 inhibition assays and molecular docking studies.

Additionally, the research group further investigated the interaction between Zishen Pills and 5-lipoxygenase (5-LOX) inhibitors [115]. It was found that 5-LOX plays a crucial role in inflammatory processes, and it is a key enzyme in the metabolism of AA to leukotriene A4 (LTA4). The research team optimized the concentration of 5-LOX enzyme, incubation conditions (temperature and time), pH, and ionic strength based on prior experiments to achieve more accurate screening results. The screening results indicated that six compounds may possess potential 5-LOX inhibitory activity, with anemarrhenasaponin I, timosaponin AI, nyasol, and demethyleneberberine demonstrating significant enzyme inhibition. Further, structure–activity relationship studies revealed that the hydroxyl group is essential for ligand binding to the 5-LOX protein, followed by the aromatic ring, which engages in π–π interactions with amino acid residues in the 5-LOX protein. This study provides a scientific foundation for the development of 5-LOX inhibitors.

## 6. Fingerprint Analysis of the Active Components of TCM

The fingerprint analysis of active ingredients in TCM is crucial for quality control and evaluation. Although traditional chemical fingerprints can reflect the overall characteristics of TCM, they are limited because the selected chemical components may not correspond directly to those that produce clinical effects. Therefore, integrating high-throughput screening technologies, such as AUF-LC-MS, to identify active ingredients in TCM and further obtain their biological fingerprints can address the limitations of chemical fingerprints and offer a novel approach for evaluating the efficacy of TCM.

Recently, Mingquan Guo’s research group [121] has made significant progress in studying Rhamnus davurica Pall. by using AUF-LC-MS. They established an AUF-LC-MS-based method to successfully screen and identify ligands in R. davurica that are potentially active against therapeutic targets like top I and COX-2 [77]. The study identified 12 potential top I ligands and 11 potential COX-2 ligands, further demonstrating that these components exhibit anti-inflammatory and anti-proliferative activities in vitro. This study not only proposes a novel method to reveal the diverse active ingredients of TCM and their potential targets but also underscores the importance of biological fingerprint analysis in TCM research.

By integrating bioaffinity technology with MS, the characteristics of active ingredients in TCM can be understood more comprehensively, providing a more scientific basis for its quality control. This approach not only addresses the limitations of traditional chemical fingerprinting but also enhances the accuracy of TCM efficacy evaluation. Future research should focus on exploring the biological fingerprints of various TCMs to advance the quality standardization and modernization of TCM, ultimately supporting its broader application in clinical practice.

## 7. Analysis of Metabolites of Small-Molecule Drugs

In the analysis of small-molecule drug metabolites, modern analytical methods are diverse and highly efficient, with LC-MS being particularly prominent. This technology not only efficiently separates and detects drugs and their metabolites but also provides detailed structural information and supports metabolic pathway research, significantly advancing the fields of pharmacokinetics and pharmacodynamics. AUF-LC-MS, a pretreatment technique, has been widely applied in drug metabolism research. This method combines ultrafiltration technology with online LC-MS analysis to rapidly and efficiently assess the metabolic rate and extent of drugs at affinity targets like liver microsomes.

Van Breemen and colleagues [122] successfully used AUF-LC-MS to evaluate the metabolic characteristics of tricyclic psychotropic drugs like promethazine and to reveal the structural features of their main metabolites. Huang et al. [99] demonstrated the potential of AUF-LC-MS in studying the pharmacological activity of natural products. They employed this technique to screen for potential lipoxygenase inhibitors in Saposhnikovia divaricata (Trucz.) Schischk. They also identified multiple metabolic pathways by using semi-preparative HPLC separation and in vitro cytochrome P450 metabolism studies, offering new approaches for evaluating the medicinal value of natural products. Methodologically, the advantage of AUF-LC-MS lies in its simplicity and high-throughput capabilities, making it particularly suitable for metabolite analysis and structural identification of complex samples. This technology not only allows researchers to quickly obtain pharmacokinetic data but also provides valuable structure–activity relationship information during drug design and optimization.

## 8. Summary and Outlook

In recent years, TCM has demonstrated unique advantages in treating complex diseases owing to its multicomponent and multitarget characteristics. Traditional methods often struggle to analyze the chemical components and pharmacological mechanisms of TCM. Bioaffinity MS offers a novel approach to address this issue. Notably, AUF-LC-MS has been widely applied in screening active ingredients in TCM owing to its high efficiency and simplicity. AUF-LC-MS shows significant potential in TCM research. This approach involves combining medicinal plant extracts with specific protein targets, using ultrafiltration to separate the conjugates, and then identifying the bound active ingredients through LC-MS. Recent studies have shown that the AUF-LC-MS yielded remarkable results in screening targets such as α-glucosidase, cyclooxygenase-2, and thrombin. This study found that compounds isolated from traditional Chinese medicine by using this method exhibited excellent enzyme inhibitory activity, with high selectivity and specificity.

Although the AUF-LC-MS method holds promising prospects for screening active ingredients in TCM, it still possesses limitations and faces various challenges. First, given the complex nature of TCM compounds, molecular interactions may compromise analytical accuracy. Future efforts might incorporate computational biology techniques to predict and confirm inter-component interactions, thereby enhancing the accuracy of screening and analysis for potentially active components. Secondly, employing multi-stage ultrafiltration membranes or a series of ultrafiltration tubes could facilitate the development of multichannel or high-throughput AUF systems, significantly enhancing the efficiency and precision of multi-target screening. Additionally, despite the therapeutic benefits of TCM volatiles like monoterpenes and sesquiterpenes, their volatile and low-density nature leads to immobilization and trapping challenges during the AUF process. Improvements might be achieved by employing tightly sealed reaction vessels, developing specialized ultrafiltration membranes, or operating in low-temperature conditions to minimize the loss of volatile components. Given the unique characteristics of volatile components, integrating auxiliary technologies such as gas chromatography for pretreatment or post-treatment might improve screening accuracy and efficiency. Moreover, false positives and nonspecific binding restrict the wider application of this technique. Utilizing enzyme denaturation controls, together with enzyme activity assays and molecular docking, can significantly mitigate nonspecific binding and boost screening accuracy.

Considering that MS and LC analysis tools have become more miniaturized and automated, the application of AUF-LC-MS is expected to become more widespread and in-depth. In the future, the use of this technology in screening TCM active ingredients should extend beyond common targets to include more significant protein targets. This approach could facilitate the discovery of new drugs and enhance the understanding of the pathogenesis of complex diseases. Therefore, AUF-MS is a powerful tool for identifying and studying the mechanisms of active ingredients in TCM. With ongoing innovations and improvements, this method is likely to play a more significant role in natural product research and new drug development. Future research should focus on overcoming current technical bottlenecks and identifying more disease-related protein targets, which would advance modern research on TCM.

## Figures and Tables

**Table 1:** Comparison between common screening methods and AUF.

Screening Technology	Features	Compare with AUF	Ref.
Traditional chemical separation methods	The traditional strategy for researching active ingredients in TCM involves “chemical extraction and separation, molecular structure identification, and pharmacological activity evaluation”.	This cumbersome operation and long cycle reduce the efficiency of active ingredient discovery.	[2]
Cell chromatography	Able to separate target molecules through specific interactions with stationary phases (e.g., resin, silica gel, etc.), suitable for applications requiring high-purity separations.	Its operation is complex and expensive, and it is not suitable for rapid separation of large-volume samples.	[40,41]
Magnetic bead adsorption screening	Different affinity ligands can be modified on the surface to improve selectivity for specific targets; separation using a magnetic field is easy to operate and does not require complex equipment.	This operation is complex and costly and is not suitable for large-scale and high-throughput processing.	[42,43]
UV-visible spectroscopy	Suitable for fast, non-destructive quantitative analysis, especially for solution samples with absorbing properties.	Its resolution and sensitivity have certain limitations, and it has certain requirements for sample concentration.	[32]
Nuclear magnetic resonance (NMR) technology	It can provide detailed information on the molecular structure and reveal the chemical environment, three-dimensional structure, dynamic behavior of molecules, etc. It can also perform quantitative analysis.	The sample concentration is required to be higher, and the instrument cost and operation difficulty are greater.	[33]
Fluorescence technology screening	Suitable for occasions requiring high sensitivity, rapid screening, and dynamic monitoring.	The instrument costs more and requires fluorescent labeling. Improper labeling may affect the results.	[34]
Electrochemical method	It is very effective for applications requiring high sensitivity, fast response, and real-time monitoring, especially for the detection of low-concentration substances.	Suitable for small-molecule analysis in liquid samples, but interference issues in complex substrates may affect data accuracy.	[35]

**Table 3:** List of active ingredients in compound preparations screened by AUF technology.

Target	Compound Preparation	Active Ingredients	Ref.
Cyclooxygenase-2	Zi-shen Pill	Twenty compounds	[114]
5-lipoxygenase	Zi-shen Pill	Six compounds	[115]
α-Glucosidase	Shenqi Jiangtang granule	Ginsenoside Rc, ginsenoside Rh1, notoginsenoside Fe, quinquenoside L10, schisandrin, isoschisandrin, gomisin D, gomisin J, pregomisin, schisantherin D	[116]
Xanthine oxidase	Mai-Luo-Ning injection	3,4-dicaffeoylquinic acid, 3,5-dicaffeoylquinic acid	[117]
Lipase	Wu-Ling-San, Ze-Xie decoction, Xiao-Xian-Xiong decoction, Xiao Chai-Hudecoction	Sixteen compounds	[118]
Augmented reality	Shenqi Jiangtang granule	Ginsenoside Rg1, Rf, Rb1, Rh1, Rd, Rg6, Rg3, Rh7, Rh2, Calycosin, Astragaloside A, Notoginsenoside Ft1, Tigloylgomisin H, Gomisin J, K3, E, Schisandrin A	[119]
Tumor necrosis factor-α	Shaoyao Gancao Fuzi Decoction	Glycyrrhizic acid, paeoniflorin, formononetin, isoliquiritigenin, benzoyl mesaconitine, glycyrrhetinic acid	[120]

## Data Availability

Not applicable.
